# Potential Role of Phenolic Extracts of *Mentha* in Managing Oxidative Stress and Alzheimer’s Disease

**DOI:** 10.3390/antiox9070631

**Published:** 2020-07-17

**Authors:** Doaa M. Hanafy, Geoffrey E. Burrows, Paul D. Prenzler, Rodney A. Hill

**Affiliations:** 1 School of Biomedical Sciences, Charles Sturt University, Locked Bag 588, Wagga Wagga, NSW 2678, Australia; dhanafy@csu.edu.au; 2 Graham Centre for Agricultural Innovation (an alliance between Charles Sturt University and NSW Department of Primary Industries), Pugsley Place, Wagga Wagga, NSW 2650, Australia; 3 Department of Pharmacognosy, National Research Centre, Dokki, Cairo 12622, Egypt; 4 School of Agricultural & Wine Sciences, Charles Sturt University, Locked Bag 588, Wagga Wagga, NSW 2678, Australia; gburrows@csu.edu.au

**Keywords:** antioxidant, apoptosis, central nervous system, heme oxygenase, mint, neurodegeneration, neuroprotection, peroxiredoxin, thioredoxin

## Abstract

With an increase in the longevity and thus the proportion of the elderly, especially in developed nations, there is a rise in pathological conditions that accompany ageing, such as neurodegenerative disorders. Alzheimer’s disease (AD) is a neurodegenerative disease characterized by progressive cognitive and memory decline. The pathophysiology of the disease is poorly understood, with several factors contributing to its development, such as oxidative stress, neuroinflammation, cholinergic neuronal apoptotic death, and the accumulation of abnormal proteins in the brain. Current medications are only palliative and cannot stop or reverse the progression of the disease. Recent clinical trials of synthetic compounds for the treatment of AD have failed because of their adverse effects or lack of efficacy. Thus, there is impetus behind the search for drugs from natural origins, in addition to the discovery of novel, conventional therapeutics. Mints have been used traditionally for conditions relevant to the central nervous system. Recent studies showed that mint extracts and/or their phenolic constituents have a neuroprotective potential and can target multiple events of AD. In this review, we provide evidence of the potential role of mint extracts and their derivatives as possible sources of treatments in managing AD. Some of the molecular pathways implicated in the development of AD are reviewed, with focus on apoptosis and some redox pathways, pointing to mechanisms that may be modulated for the treatment of AD, and the need for future research invoking knowledge of these pathways is highlighted.

## 1. Development of Alzheimer’s Disease

Alzheimer’s disease (AD) is a progressive neurodegenerative disorder that disrupts the normal brain structure and function significantly, leading to memory loss [1]. The incidence of developing AD increases with age, especially after 65 years [2]. Dementia is a syndrome that describes the progressive deterioration of intellectual function. AD is the most common type of dementia [2].

The 2019 World Alzheimer Report estimates that, worldwide, there are more than 50 million people living with dementia and this number is expected to increase to 152 million by 2050, as populations age [3]. A review of AD incidence data reveals that in the United States, AD is listed as the sixth-leading cause of death. An estimated 5.8 million Americans of all ages were living with Alzheimer’s dementia in 2019 (about 1.7%) [4]. In Australia, nearly 459,000 people (about 1.8%) are living with dementia, and this number is expected to increase to an estimated 590,000 by 2025 and 1,076,000 by 2050. Dementia is the second leading cause of death in Australia [5]. Statistics about the disease are very limited in the Middle East [6]. In Egypt, the percentage of AD and other types of dementia among people over the age of 60 years in the Assiut governorate in Upper Egypt is 4.5% [7], and that in the Sharkia governorate in the Nile Delta is 3.66% [8]. The prevalence of dementia in 2019 was 5.6% and 1.5% in China and Canada, respectively [9,10]. The prevalence of dementia varies greatly across the world, like any other disease [11]. This is partially due to the lack of methodological uniformity among studies, including diagnostic criteria and different mean population ages, or due to the different socioeconomic burdens and educational backgrounds [11]. It is expected that three-quarters of the world’s population aged 60 years or over will be living in developing countries by 2025 [11].

An early symptom of AD is a worsening ability to remember new information due to a loss or malfunction of neurons [1]. As damage spreads, patients experience other difficulties, such as a loss of memory and language impairment, challenges in performing familiar tasks, difficulties in planning or solving problems, trouble understanding visual images and spatial relationships, confusion with place or time, misplacing items and losing the ability to retrace steps, poor judgment, detachment from work or social activities, and changes in mood and personality, until there is a complete dependence on a carer [4].

AD develops from multiple causes [1], and several hypotheses exist to explain the neurodegenerative process [12]. The accumulation of abnormal proteins, amyloid-β (Aβ) deposits (senile plaques), outside the neurons and the hyperphosphorylated tau protein (neurofibrillary tangles) inside the neurons, are the major hallmarks of AD, along with synaptic and neuronal loss [2]. Cells normally release soluble Aβ after the amyloid precursor protein (APP) is cleaved. AD involves abnormal APP cleavage by β- and γ-secretases that leads to the deposition of Aβ into dense beta sheets and the formation of senile plaques [1,13]. Tau is a protein that stabilizes microtubules in neurons [14]. The abnormal post-translational modification of the tau protein, particularly by phosphorylation, induces conformational changes and the aggregation of tau, resulting in the formation of neurofibrillary tangles [14]. The status of tau phosphorylation is an indicator of the abnormal activity of kinases and phosphatases during the progression of the disease, and increased phosphorylation in the cerebrospinal fluid might help in differentiating AD from other types of dementia [14]. Abnormally low or high levels of Aβ oligomers and hyperphosphorylated tau proteins can induce impaired synaptic plasticity and cause pre- and post-synaptic dysfunction and deterioration in AD [14]. However, it is still not completely understood whether Aβ accumulation is one of the causes of AD or if it is a consequence of neuronal damage caused by noxious stimuli that lead to its increased production or reduced clearance [15]. It was also found that oxidative stress occurs early in AD pathology, as elevated signs of oxidative stress have been found in AD brain tissue samples [12,16]. Excess reactive oxygen species (ROS) are implicated as initiators of degenerative processes and have been linked to neurodegenerative diseases and ageing [17]. Aβ induces oxidative stress that is reflected by lipid peroxidation, protein and DNA oxidation, free radical formation, and neurotoxicity [12,18]. The products of lipid peroxidation, such as 4-hydroxynonenal, can also enhance γ-secretase activity and the generation of Aβ plaques in neurons [19]. Disturbances in the levels of copper, zinc, and iron (metal dyshomeostasis) were found in AD brains and have been linked to the pathology of the disease [20]. These metals can induce the formation of ROS and Aβ oligomers [20]. It is also suggested that mechanisms leading to the activation of immune mediators are implicated in AD pathology [2]. Recent studies suggest an infectious basis for AD, and that intracerebral infection by certain pathogens may induce Aβ fibrillization as an antimicrobial defense mechanism, resulting in Aβ deposition [2]. A genetic risk factor (apolipoprotein E (APOE)) could be linked to the increased deposition and decreased clearance of Aβ [2].

The overstimulation of glutamate NMDA (N-methyl-D-aspartate) receptors produces excitotoxic effects on neurons that can result in neurodegeneration or apoptosis [12]. The activation of inflammatory pathways was observed in the brains of individuals with AD (neuroinflammation) [21]. Furthermore, alteration in acetylcholinesterase (AChE) and butyrylcholinesterase (BuChE) and the abnormal acetylation of histone were involved in the pathophysiology of AD [1,22]. Histone deacetylases (HDAC) comprise a family of enzymes that catalyze the hydrolysis of acetyl groups from an N-acetyl lysine amino acid on a histone, which allows the histones to wrap the DNA more tightly [23]. Histones are the building blocks of chromatin at which transcriptional regulation occurs. The post-translational modification of histones, such as methylation, phosphorylation, and acetylation, affects transcriptional regulation [23]. Insulin resistance might also be associated with AD pathology, as impaired neurotrophic effects of insulin were reported in aged brains, which reduced the ability of the brain to repair neuronal damage, resulting in synaptic, metabolic, and immune response dysfunctions [15]. Mitochondrial dysfunction also plays an important role in AD pathology and progression [24]. The reduced expression of mitochondrial enzymes and oxidative phosphorylation genes with altered mitochondrial structure, potential, and permeability were observed in AD brains [17]. Increased mitochondrial membrane permeability leads to the excessive production of ROS and leakage of proteins, which initiates a cascade of reactions leading to cell death [17,24]. The accumulation of Aβ plaques and tau tangles was linked to mitochondrial impairment in AD; however, there is still a debate whether they are the cause or a consequence of mitochondrial dysfunction [24].

Although there are a number of controversial hypotheses and factors used to explain the initiation and progression of AD, neuronal death is the common factor. As discussed above, oxidative stress plays a pivotal role in the disease development and can exacerbate key events in AD. Thus, this review highlights the signaling cascade of apoptosis and some cellular antioxidant defense mechanisms and how mint extracts can alter these systems towards a potential AD treatment.

## 2. Cellular and Molecular Pathways Implicated in AD

### 2.1. Apoptosis and AD

**Figure 1:**
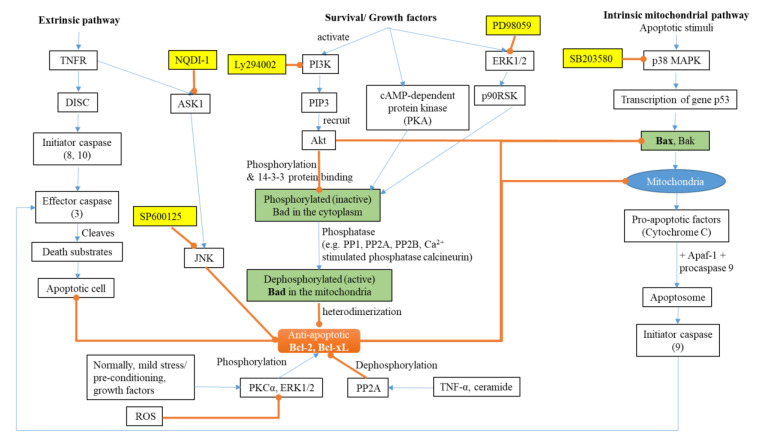
A simplified diagram of the signaling pathways of apoptosis. Akt (protein kinase B); ASK1 (apoptosis signal-regulating kinase 1); Apaf-1 (apoptotic protease activating factor 1); Bad (Bcl-2-associated death promoter); Bak (Bcl-2 homologous antagonist killer); Bax (Bcl-2-associated X protein); DISC (death-inducing signaling complex); ERK1/2 (extracellular signal-regulated kinases, 1 and 2 isoforms); JNK (c-Jun NH_2_-terminal kinases); p90^RSK^ (p90 ribosomal S6 kinase); PI3K (phosphatidylinositol 3-kinase); PIP3 (phosphatidylinositol trisphosphate); PKCα (protein kinase C α); PP2A (protein phosphatase 2A); TNF-α (tumour necrosis factor α); TNFR (tumour necrosis factor receptor); ROS (reactive oxygen species); → (activation); ⊸ (inhibition). Compounds in yellow boxes are inhibitors.

Neuronal death from apoptosis has been found in the brains of patients suffering from neurodegenerative diseases. Apoptotic cell death is characteristic in neurons and glial cells in AD [25]. Apoptosis is a genetically programmed cell death (PCD). There are two pathways of PCD: the stimulation of death receptors by external ligands (extrinsic pathway) and an internal mitochondrial pathway (intrinsic pathway) (Figure 1). Both pathways lead to the recruitment and activation of initiator caspases (that transmit the initial apoptotic signals), then effector caspases (responsible for the final phase of cell death) [26]. The morphological characteristics of an apoptotic cell include chromatin condensation, DNA fragmentation, nuclear fragmentation, and membrane blebbing [27].

Caspases (cysteine-aspartic proteases) are a family of protease enzymes that play the major role in apoptosis. Caspases have a cysteine protease activity, cleaving a target protein after an aspartic acid residue, leading to changes in the structure (degradation) and function of the protein. A cascade involving the activation of about nine different caspases is involved in the stimulation of the pro-apoptotic pathway [26]. The caspase cleavage of APP and tau may promote the formation of Aβ and neurofibrillary tangles [28]. Therefore, preventing the activation and execution of apoptosis may provide an effective means of treating AD [28].

The extrinsic pathway is activated when death receptors, such as the tumour necrosis factor receptor (TNFR), are stimulated, then activating the death-inducing signaling complex (DISC). The DISC, in turn, activates initiator caspase—e.g., caspase 8—leading to the activation of effector caspases—e.g., caspase 3 [26].

The intrinsic pathway can be initiated by apoptotic stimuli, such as DNA damage, chemotherapeutic agents, ROS, the removal of survival factors (such as cytokines and hormones), and ageing. The cascade starts when apoptotic stimuli activate the p53 protein, which activates p21 and the pro-apoptotic members of the Bcl-2 protein family (Bax (Bcl-2-associated X protein) and Bak (Bcl-2 homologous antagonist killer)). Pro-apoptotic proteins form pores in the mitochondria, affecting the outer membrane permeability and inducing the release of cytochrome C from the mitochondria. Cytochrome C binds with apoptotic protease activating factor 1 (Apaf-1) to form an apoptosome. The apoptosomes then recruit and activate the inactive procaspase 9. Once activated, this initiator caspase can then activate effector caspases and trigger a cascade of events that lead to apoptosis [26,27]. This process is still incompletely understood, although it has been studied extensively [27] (Figure 1).

The process of apoptosis is regulated through the anti-apoptotic members Bcl-2 and Bcl-xL, which inhibit the mitochondrial pathway and prevent apoptosis. Anti-apoptotic proteins antagonize the effects of the pro-apoptotic proteins and prevent the release of cytochrome C. It was found that the level of both the pro-apoptotic and anti-apoptotic proteins is high in AD brains [29]. The rise in the level of the anti-apoptotic proteins could be a compensatory response in AD to protect the remaining neurons from apoptosis [29]. Inhibiting apoptosis might prevent or treat a wide range of common degenerative disorders, but no successful treatment has been developed to date [26].

The Bcl-2-associated death promoter (Bad) protein is another protein that can indirectly promote apoptosis through the intrinsic mitochondrial pathway [30]. Active Bad heterodimerizes with anti-apoptotic proteins, preventing their action; hence, Bad is considered a pro-apoptotic protein. The phosphorylation of Bad by protein kinase B (Akt) causes its inactivation. The phosphorylation of Bad induces a conformational change in Bad, which allows binding with the 14-3-3 protein, which sequesters Bad in the cytoplasm and prevents its interaction with Bcl-2 or Bcl-xL. The dephosphorylation of Bad by phosphatases such as Ca^2+^-stimulated phosphatase calcineurin and protein phosphatase 2A (PP2A) drive its activation through its release from sequestration [30].

### 2.2. The Redox System and AD

ROS are generated normally through cell metabolism. At physiological concentrations, ROS perform normal functions, such as the induction of mitogenic response, the regulation of signal transduction, and involvement in protection against infectious agents [31]. Under normal conditions, the production of ROS is balanced by the antioxidant system. Imbalance by the over-production of ROS and/or insufficient antioxidants leads to oxidative stress. Excessive ROS can cause cell membrane damage through lipid peroxidation; the modification of signal and structural proteins, leading to misfolding and aggregation; and the oxidation of RNA/DNA, which interrupts transcription and results in gene mutation [31]. Oxidative stress can change the expression and activities of enzymes interacting with different signaling pathways [16]. As the brain is the organ in which the consumption of oxygen occurs at the highest rate, it is highly susceptible to oxidative stress, leading to neurodegenerative diseases [31]. Impaired natural antioxidant defense systems were identified in AD patients [20]. Glutathione is one of the most prevalent cellular antioxidants and exists in reduced (GSH) and oxidized (GSSG) forms [24]. Glutathione peroxidase catalyzes the oxidation of GSH into GSSG, while glutathione reductase catalyzes the reverse reaction [24]. The reduction in H_2_O_2_ is coupled to the oxidation of GSH [20]. Under conditions of oxidative stress and in the brains of AD patients, a decreased GSH/GSSG ratio was detected, which indicated the altered antioxidant ability of the GSH system [20,24]. It was also found that the severity of the disease is proportional to the magnitude of the changes in the GSH redox cycle [24]. Superoxide dismutase (SOD) and catalase (CAT) are other cellular antioxidant enzymes that catalyze the conversion of superoxide into H_2_O_2_ and then into water, respectively [24]. The decreased activity of these enzymes was observed in cognitively impaired patients [24]. Altered expression levels of the antioxidant proteins, thioredoxin, peroxiredoxin, and heme oxygenase, were also reported in AD brains [24] and are covered in more detail in this review.

At normal physiological concentrations, ROS are key components of cellular signaling cascades for controlling cell proliferation, survival, and migration. The phosphorylation of Bad via Akt inhibits the pro-apoptotic cascade by making the anti-apoptotic proteins available to exert their action (Figure 1). PP2A dephosphorylates Akt at threonine 308 and serine 473, thus blocking the phosphatidylinositol 3-kinase (PI3K)/Akt pathway. H_2_O_2_ can inactivate PP2A at the cysteine site and therefore activates the Akt pathway, which facilitates cell survival [31].

Mitogen-activated protein kinases (MAPK) are a family of serine/threonine kinases. There are three major MAPK pathways that induce several cellular functions, such as gene expression, mitosis, and apoptosis, through the phosphorylation of the specific serine and/or threonine residues of target proteins: p38 MAPKs (including p38α, p38β, p38γ, and p38δ isoforms), c-Jun NH_2_-terminal kinases (JNK, including JNK1, JNK2, and JNK3 isoforms), and extracellular signal-regulated kinases (ERK, including ERK1 and ERK2 isoforms). MAPK kinase kinase (MAPKKK) phosphorylates and thereby activates MAPK kinase (MAPKK), which, in turn, phosphorylates and activates MAPK, which regulates cell proliferation [32]. ROS can activate the ERK signal [31], which downstream activates p90^RSK^ (p90 ribosomal S6 kinase), which mediates the binding of Bad to 14-3-3 and prevents Bad-mediated cell death [33]. Generally, the activation of ERK1/2 promotes cell survival, but under certain conditions, ERK1/2 can have pro-apoptotic functions [34].

Apoptosis signal-regulating kinase 1 (ASK1) is a member of the MAPKKK family that is activated by oxidative stress induced by ROS, tumour necrosis factor (TNF) α, lipopolysaccharide, endoplasmic reticulum stress, and calcium influx; it selectively activates the JNK and p38 MAPK pathways [32]. Oxidative stress activates ASK1, which activates JNK and p38 MAPK and decreases the activity of PP2A, which leads to tau hyperphosphorylation, one of the major hallmarks of AD brains [31]. Additionally, the activation of JNK and p38 stimulates β-secretase, causing Aβ accumulation and neuronal cell death [31,35].

#### 2.2.1. Thioredoxin and Peroxiredoxin

**Figure 2:**
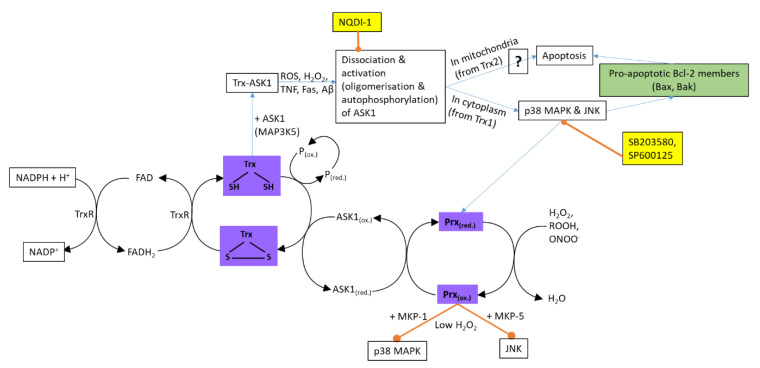
A simplified diagram of the regulation of ASK1-mediated apoptosis by thioredoxin (Trx) and peroxiredoxin (Prx). Aβ (amyloid β); ASK1 (apoptosis signal-regulating kinase 1); FAD (flavin adenine dinucleotide); Fas (apoptotic receptor); JNK (c-Jun NH_2_-terminal kinases); MKP (MAPK phosphatases); NADP (nicotinamide adenine dinucleotide phosphate); P (protein); TNF (tumour necrosis factor α); ROS (reactive oxygen species); TrxR (thioredoxin reductase); **?** (means that this pathway is still unexplored); → (activation); ⊸ (inhibition). Compounds in yellow boxes are inhibitors.

The thioredoxin (Trx) family proteins are oxidoreductases that act as cofactors in the activity of other enzymes and facilitate the reduction of several transcription factors and signaling molecules [36]. Mammalian genomes encode two Trx systems: Trx1 and TrxR1 (thioredoxin reductase 1) present in the cytosol and Trx2 and TrxR2 in the mitochondria [37]. Trx1 contains two redox-active cysteine residues in their conserved active centres that can be oxidized to form intramolecular disulphide bonds (Trx-S2) (Figure 2). The reduction of Trx-S2 is catalyzed by TrxR, with NADPH (nicotinamide adenine dinucleotide phosphate) as the electron donor. The thiol oxidoreductase activity of Trx makes it suitable to reduce protein disulphides and H_2_O_2_, thus functioning as an electron carrier for the catalytic actions of peroxidases and as a protector protein against oxidant-mediated disulphide bond formation [38]. Reduced Trx1 can bind to the N-terminal domain of ASK1, which inhibits its kinase activity, preventing cell apoptosis [32]. Thus, Trx1 can protect against oxidative stress and prevent cell apoptosis by removing H_2_O_2_. ROS, TNFα, and Aβ oxidize Trx, resulting in the dissociation of the Trx-ASK1 complex and the activation of ASK1, which induces apoptosis via the JNK and p38 MAPK pathways [37,39]. ASK1 also induces a mitochondria-dependent apoptotic pathway, but this pathway is not well understood [39]. The treatment of SH-SY5Y cells with Aβ resulted in the oxidation of Trx1 [37]. The over-expression of Trx1 in SH-SY5Y cells and in rat primary hippocampal neurons resulted in a protective effect against Aβ-induced cell death [37]. It was found that Trx1 protein levels were decreased in patients during amnestic mild cognitive impairment and in several regions of AD-affected brains [40,41,42]; however, in another study, there was no significant difference in the Trx levels between the brains of control and AD patients, although the TrxR1 activity was enhanced in the AD patients [37].

Peroxiredoxins (Prx) are a family of thioredoxin peroxidase enzymes, and their activity is linked to Trx [36]. There are six Prxs in mammalian cells that play important roles in the response to H_2_O_2_ [37]. Prxs are upregulated by stress agents such as H_2_O_2_. Prxs are able to reduce H_2_O_2_ to H_2_O; meanwhile, the peroxidatic cysteine becomes oxidized to a sulphenic acid. The sulphenic acid then reacts with the cysteine of the adjacent Prx molecule, forming an intermolecular disulphide bond. The resulting Prx disulphides are reduced by Trx using electrons from NADPH [36]. Prx can also catalyze the oxidation of ASK1 in response to peroxide, forming the Prx-ASK1 disulphide complex, resulting in p38 phosphorylation [43]. Altering the expression of Prx1 or Prx2 had different effects; a decrease in the Prx1 expression with an increase in Prx2 inhibits the activation of ASK1/p38 induced by H_2_O_2_ [37]. The signaling pathways that regulate the expression of Prx are not clear [44]. The overexpression of Prx1 in PC12 cells and primary neurons attenuated Aβ-induced toxicity and protected dopaminergic neurons from apoptosis [37]. Additionally, the overexpression of Prx2 protected against Aβ-induced toxicity in a transgenic mouse model for AD [37]. The expression levels of Prx1, Prx2, and Prx6 were high in the brains of AD patients, while that of Prx3 was low [45,46,47]. The redox states of Prx2 and Prx6 in the serum and brains of AD patients were more oxidized [37].

#### 2.2.2. Heme Oxygenase-1 (HO-1)

**Figure 3:**
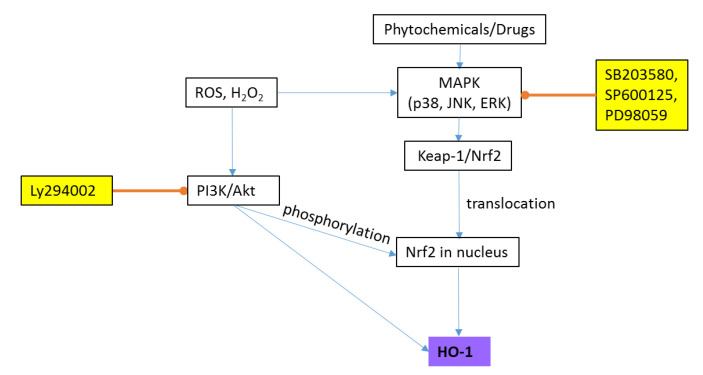
A simplified diagram of the signaling pathway leading to heme oxygenase-1 (HO-1) expression. Akt (protein kinase B); ERK (extracellular signal-regulated kinases); JNK (c-Jun NH_2_-terminal kinases); Keap-1 (kelch-like ECH-associated protein-1); Nrf2 (nuclear factor (erythroid-derived 2)-like 2); PI3K (phosphatidylinositol 3-kinase); ROS (reactive oxygen species); → (activation); ⊸ (inhibition). Compounds in yellow boxes are inhibitors.

Heme oxygenase (HO) is an enzyme responsible for the catalytic degradation of heme into biliverdin, ferrous ions, and carbon monoxide. There are two forms of HO: HO-1 (an inducible form) and HO-2 (a constitutively expressed form) [48]. HO-1 can be expressed by a variety of stressors, including H_2_O_2_. HO-1 has antioxidant, anti-inflammatory, anti-apoptotic, and antiproliferative properties. Non-toxic phytochemicals (e.g., curcumin), polyphenols, and drugs stimulate the expression of HO-1 through the three major MAPK pathways: ERK, JNK, and p38 MAPK [48] (Figure 3). HO-1 can also be upregulated through the PI3K/Akt pathway [49]. HO-1 is highly inducible under oxidative stress through nuclear factor (erythroid-derived 2)-like 2 (Nrf2). Nrf2 is found in the cytoplasm bound to its inhibitor kelch-like ECH-associated protein-1 (Keap-1), which leads to its ubiquitination and degradation under normal conditions [48]. Oxidative stress stimulates the dissociation of Nrf2 from Keap-1 and its translocation into the nucleus, where it mediates HO-1 gene transcription [50]. Preconditioning SH-SY5Y cells with a low concentration of H_2_O_2_ before inducing cell injury with a higher concentration protected the cells against this injury through the up-regulation of HO-1 via the PI3K/Akt pathway [49]. The overexpression of HO-1 with an increased serine phosphorylation on the HO-1 protein was found in AD, suggesting that it may be subjected to oxidative damage and undergo post-translational modifications in brain tissue [50].

As AD pathology occurs years before its diagnosis, the process of apoptosis might be different from the classical one [29]. Although the apoptotic and redox pathways have been identified to be involved in the events of AD development and progression, little is known about the predominant apoptosis-regulating factors and oxidative stress, along with the antioxidant defence mechanism that underlies the process in AD brains. The upstream and downstream kinases seem to play a pivotal role in the signaling cascades. The elucidation of the mechanistic basis for the protection of the cells from oxidative damage and suppressing cell death or enhancing cell survival might be useful aspects in the treatment of the disease, and, therefore, are worth further investigation.

## 3. Treatment of AD

No pharmacotherapeutics have yet been able to cure patients with AD [2]. Memory deficits in AD patients results from a defect in the cholinergic system [51]; hence, an important approach to treat AD is to enhance the levels of the neurotransmitter acetylcholine (ACh) in the brain. This can be achieved by the inhibition of cholinesterase (ChE), the enzyme responsible for the hydrolysis of ACh [52]. Approved medications are three AChE inhibitors—rivastigmine, galantamine, and donepezil—and one NMDA (glutamate) receptor antagonist—memantine [2]. These approved medications are only symptomatic and palliative treatments [1]. Many compounds have entered clinical trials in the last few years, with none being efficacious, and in some cases treatments have resulted in the further deterioration of cognitive function [15]. The failure of these trials could be due to the heterogeneous nature, together with the incomplete understanding of the pathophysiology of AD [53]. It was recently suggested to combine a number of therapeutic agents that target different pathogenic mechanisms, but this approach faces some challenges (e.g., the proper selection of doses and drug interactions) [53].

Medicinal plants contain a variety of secondary metabolites that provide a promising source for improving cognitive performance and the treatment of neurodegenerative and age-related diseases [54,55]. Traditional uses of medicinal plants for improving memory have led to drug development. *Galanthus nivalis* L. (Amaryllidaceae) has been used traditionally in Bulgaria and Turkey for the treatment of neurological disorders [56]. This traditional use was the key for performing further studies on this plant, resulting in the isolation of the alkaloid, galantamine [57]. Researchers found that galantamine is an acetylcholinesterase inhibitor, and it was approved in 2004 by the Food and Drug Administration (FDA), USA, as a medication for AD [58], which emphasizes the importance of ethnopharmacology in drug discovery.

Moreover, the adverse effects of synthetic drugs can lead to their discontinuation. For example, tacrine (brand name “Cognex”) was the first centrally acting AChE inhibitor (approved for the treatment of AD in 1993), but was discontinued in 2013 [59] because of its hepatotoxic effect [60].

## 4. History and Uses of *Mentha*

*Mentha* (commonly known as mint) is a genus in the Lamiaceae, with 18 species and 11 hybrids [61] distributed in diverse environments mainly in temperate and sub-temperate regions across the world [62]. *Mentha* has a long history of cultivation and is widely known for its culinary, medicinal, and aromatherapeutic properties. Since ancient Egyptian times, peppermint has been used to treat indigestion. The ancient Greeks and Romans also used it for soothing the stomach [63]. In the Middle Ages, peppermint was used as a tooth polisher and to keep rats and mice out of storerooms because of its strong smell. Peppermint then became popular in the eighteenth century in Western Europe as a folk remedy for nausea, vomiting, morning sickness, menstrual disorders, and respiratory infections [64]. In 1721, it was listed in the London Pharmacopoeia as a remedy for treating a wide range of ailments including sores, venereal disease, colds, and headaches [64].

Mint was used in Asia and the Mediterranean region for the treatment of gastric and intestinal colic and spasms [65]. Different *Mentha* taxa have several ethnomedicinal uses (Table S1). For example, *M. pulegium* was used in Greece and the Mediterranean region for the treatment of dizziness, nausea, sea sickness, headache, migraine, intestinal pains or inflammation, and toothache. It was also used as an anti-convulsive, anesthetic, sedative, and spasmolytic [66].

Mints are edible plants known for their refreshing taste and aroma. *M. pulegium* was commonly used in Portugal as a spice in traditional food preparations [67]. *M. spicata* and *M.* × *piperita* are used as teas in the Mediterranean region, while their dry or fresh leaves are used as a flavor enhancer [68,69].

Mints have a history of uses, implicating activity within the central nervous system (CNS) in traditional medicine. In South Africa, the dried leaves of *M. aquatica* are burned and the smoke inhaled for the treatment of mental illnesses and for protection against and removal of curses and evil spirits, which resemble symptoms of depression [70]. In Mediterranean countries, *M. arvensis* and *M.* × *piperita* are used to treat neuralgia [66,71], *M. pulegium* and *M. suaveolens* are used as anticonvulsives, and *M. longifolia*, *M. spicata* and *M.* × *villosa-nervata* are used as sedatives [66].

## 5. Chemical Composition and General Bioactivities of *Mentha*

Essential oils (EO) and polyphenols are considered the main chemical groups responsible for the broad range of bioactivities of *Mentha* [72]. The EO of *Mentha* include monoterpenes and sesquiterpenes, the content of which varies from species to species, in addition to the terpenoids, menthol, menthone, neomenthol, menthyl acetate, isomenthone, 1,8-cineole, linalool, α-pinene, β-pinene, limonene, carvone, and pulegone [73] (Table S2, Figure S1).

The essential oils of mints are commercially important for the pharmaceutical, food, and cosmetic industries. They are often used as flavoring agents in toothpaste, candy canes, chewing gums and beverages [73]. They are commonly used for their cooling, invigorating, and anesthetic qualities. They have been described as being effective in treating a wide range of gastrointestinal tract complaints [73]. *Mentha* EO have been reported to have anti-inflammatory, hepatoprotective, hypotensive, vasorelaxant, antioxidant, analgesic, antibacterial, antifungal, antiviral, cytotoxic, and insecticidal properties [73].

The phenolic composition of mint extracts has recently gained the attention of researchers, especially after finding that their bioactivities are highly associated with their phenolic content [69,74]. It is believed that the phenolic compounds are the main active ingredients of, for example, mint teas, because they are prepared uncovered, so the volatile substances are lost in the process [75]. Phenolic acids and flavonoids are the main phenolic classes identified in mints [73]. Rosmarinic acid is a characteristic compound of *Mentha*, being present in almost all species [73]. The main phenolic compounds reported in mints are luteolin, naringenin, apigenin, hesperitin, eriodictyol, and their glycosides, as well as rosmarinic, caffeic, chlorogenic, and salvianolic acids and their derivatives [73] (Table S2, Figure S2).

It has been suggested that 75% of the phenolic compounds present in a mint infusion may be responsible for its biological activities [76]. A strong correlation between the total phenolic content (TPC) of mints and their antioxidant capacity has been reported [69,74,77], indicating that the biophenols may be responsible for the antioxidant activity. Conforti et al. [78] and [79] found that a hydroalcoholic extract of *M. aquatica* which showed antiproliferative activity in breast cancer cells in vitro and free radical scavenging and antioxidant activities contained high levels of phenolics. The flavonoids (apigenin and luteolin) and their glucosides (rutinoside and glucuronide) isolated from *M. longifolia* subsp. *longifolia* showed antimutagenic activity in vitro [80,81]. Peppermint teas that showed antichlamydial activity had a high content of luteolin and apigenin glycosides [82].

Water-soluble extracts from *M. aquatica*, *M. haplocalyx*, *M.* × *dalmatica*, *M.* × *verticillata*, *M. arvensis* var. *japanensis*, *M. spicata* var. *crispa* and *M.* × *piperita* ʻʼFrantsilaʼ, *M.* ʻMoroccoʼ and *M.* ʻNative Wilmetʼ species and cultivars showed antioxidative properties, and the level of activity was strongly correlated with the phenolic content [83].

## 6. Bioactivities of *Mentha* Extracts Related to CNS, Oxidative Stress and AD

EO of *M. suaveolens* and *M. pulegium* may have an effect on the CNS showing AChE inhibitory activity [67,84]. *M. aquatica* EO have an affinity for GABA-benzodiazepine receptors, indicating sedative or anticonvulsive activities [85]. Moreover, the EO of *M.* × *piperita* were reported to enhance memory in healthy participants [86].

Dietary flavonoids and phenolic acids have been reported to protect neurons from injury and promote memory, learning, and cognitive function, indicating a neuroprotective effect [87]. For example, the flavonoid quercetin, which is generally found in fruits and vegetables, has a neuroprotective activity against agent-induced toxicity and enhances the resistance of neurons to oxidative stress and excitotoxicity through a modulation in the mechanisms of cell death in vitro [88]. The flavonoids gallocatechin gallate and theaflavin inhibit Aβ aggregation, and the stilbenes, resveratrol and its glucoside, could suppress the aggregation [89]. Quercetin is also reported to ameliorate AD pathology and protect cognitive and emotional function in aged triple-transgenic AD-model mice [88].

**Figure 4:**
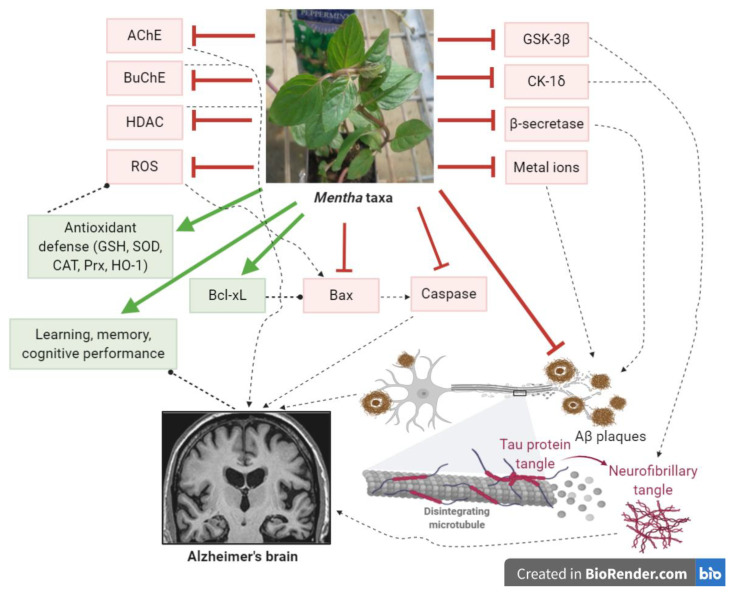
Theoretical scheme of possible mechanisms underlying the neuroprotective effect of *Mentha* taxa.

Extracts of different *Mentha* taxa containing a number of flavonoids and/or phenolic acids showed a wide range of activities relevant to AD (Figure 4). The extracts of 19 *Mentha* taxa rich in phenolic compounds showed antioxidant and HDAC inhibition activities, which were strongly associated with their biophenol content [74]. The extracts also exhibited AChE and BuChE inhibition activities [74]. The ethanolic extracts from *M. longifolia*, *M. pulegium*, and *M.* × *piperita* and their phenolic constituents, rosmarinic, caffeic, chlorogenic, and ferulic acids, showed antioxidant and AChE inhibitory activities, with rosmarinic acid highly likely to contribute to the activities of the extracts [54]. The methanolic extracts of the above species and *M. aquatica* and *M. suaveolens* showed antioxidant and lipid peroxidation inhibition activities [73], as well as a monoamine oxidase-A (MAO-A) inhibition activity, indicating an antidepressant effect [90]. *M. piperita* 70% methanol extract containing flavones and phenolic acids inhibited β-secretase (the enzyme involved in the formation of Aβ), glycogen synthase kinase (GSK-3β), and casein kinase 1δ (CK-1δ) (the enzymes involved in the pathological accumulation of tau) [91]. The methanolic extracts of *M.* pulegium, *M.* rotundifolia, *M.* villosa, *M.* arvensis, *M. aquatica*, and *M.* piperita showed antioxidant and metal ion chelating ability [92]. The ethanolic extract and water infusion of *M. longifolia* var. *calliantha* showed antioxidant and AChE inhibitory activities and moderate metal chelating activities [93]. The methanolic extracts of *M. aquatica*, *M. longifolia*, and *M.* × *piperita* also showed an affinity to the GABA-benzodiazepine receptor, indicating an anxiolytic effect [90]. Extracts rich in phenolic compounds of *M. australis*, *M. diemenica*, *M. spicata* var. *crispa*, *M.* × *piperita*, *M.* × *piperita* var. *officinalis*, and *M. requienii* inhibited β-secretase and Aβ aggregation in vitro [94]. *M. aquatica* and *M.* × *piperita* had a neuroprotective effect against the H_2_O_2_-induced toxicity in pheochromocytoma (PC12 cells) [90]. *M. suaveolens* aqueous leaf extract reduced the generation of intracellular ROS in H_2_O_2_-induced damage in HaCaT keratinocytes, decreased the levels of H_2_O_2_-induced apoptotic genes (initiator caspase (caspase-9), effector caspase (caspase-3), and poly (ADP-ribose) polymerase) and induced the expression of HO-1 through the translocation of Nrf2 upon H_2_O_2_ exposure [95]. *M. diemenica* and *M. requienii* extracts lowered the caspase-3/7 activity in H_2_O_2_-induced damage in SH-SY5Y cells, indicating a neuroprotective effect [94]. Pretreating SH-SY5Y cells with *M. diemenica* extracts resulted in a downregulation of the expression of the pro-apoptotic protein, Bax, and the upregulation of the anti-apoptotic protein, Bcl-xL, and the antioxidant genes, Prx and HO-1, indicating its potential ability to suppress apoptosis and protect against oxidative stress [94].

Freeze-dried extracts of *M. piperita* were reported to have protective effects in mice against Aβ formation, ageing-induced stress, amnesia, and neurodegeneration [96]. The extract was also reported to improve acquisition and retention in both exteroceptive and interoceptive behavioral models, and to reverse amnesia and inhibit decreases in ACh levels in the brains of ageing and scopolamine-induced amnesic mice [96]. The oral administration of *M. piperita* and *M. arvensis* aqueous extracts enhanced the activity of antioxidant defenses (GSH, SOD, or CAT) in the brain tissues of gamma-irradiation- and haloperidol-induced oxidative stress in mice, respectively [97,98]. *M. spicata* extract with 5% rosmarinic acid was reported to have beneficial effects on learning and memory and the brain tissue markers of oxidation [99]. A dried aqueous *M. spicata* extract containing high levels of rosmarinic acid (≥14.5%) improved working memory and cognitive performance in individuals (50–70 years of age) with age-associated memory impairment in a randomized, double-blind, placebo-controlled study [100].

**Figure 5:**
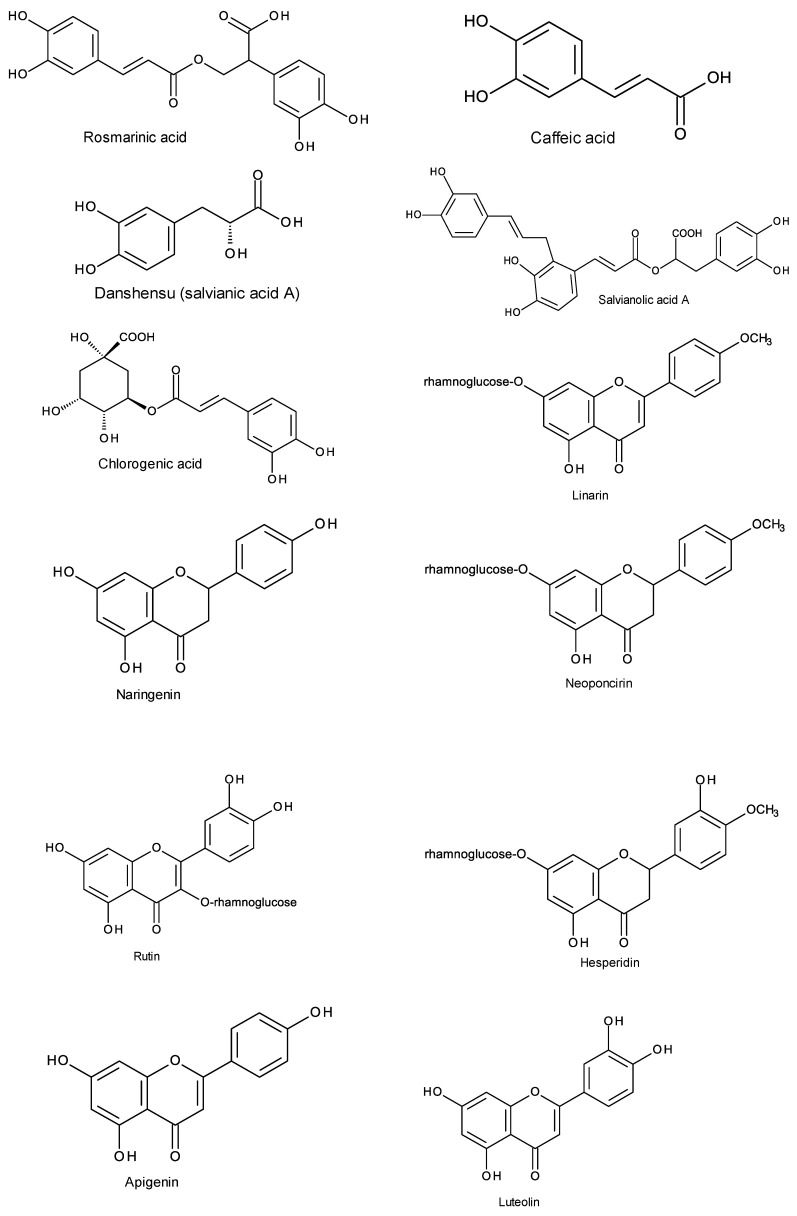
Chemical structure of some phenolic compounds with reported bioactivities relevant to AD identified in the *Mentha* taxa.

Many phenolic compounds identified in *Mentha* taxa (Table 1 and Figure 5) have been reported to have AD-related bioactivities. For example, linarin (acacetin-7-*O*-β-D-rutinoside) isolated from a flower extract of *M. arvensis* showed AChE inhibitory effects [101]. It also had neuroprotective effects against Aβ25-35-induced neurotoxicity in cultured rat PC12 cells through the activation of the PI3K/Akt pathway [102]. The ethanolic extract of the aerial parts of *M. aquatica* and the isolated flavanone, naringenin, showed affinity for the GABA-benzodiazepine receptor, suggesting an anti-convulsive activity or a sedative effect [85]. Naringenin isolated from a 70% ethanol extract of *M. aquatica* leaves inhibited MAO-A and MAO-B, indicating its antidepressant and anti-Parkinson’s disease effects, respectively [70]. Naringenin can pass the blood-brain barrier, so it can have an effect on the CNS [85]. Caffeic acid was reported to protect mice from the neuronal loss and memory impairment induced by focal cerebral ischaemia, which includes a complex cascade of events such as inflammation and oxidative stress [103]. Salvianolic acid A was reported to have a neuroprotective effect against acute ischemic stroke in mice, as it increased their survival rate, enhanced their activity levels, and improved the severity of brain infarction and apoptosis [104]. Rosmarinic acid showed a neuroprotective effect against H_2_O_2_-induced neurotoxicity in SH-SY5Y cells [105] and against Aβ-induced damage in PC12 cells [106]. Hamaguchi et al. [107] found that rosmarinic acid prevented the development of AD pathology by decreasing the Aβ deposition in the brains of AD model transgenic mice (Tg2576). Danshensu (salvianic acid A) was reported to improve cognitive performance in an animal model of AD [108] and protect PC12 cells against neurotoxicity [109]. Danshensu also increased the expression of HO-1 and suppressed 6-hydroxydopamine-induced oxidative damage via the PI3K/Akt/Nrf2 signaling pathway [110]. Rutin (quercetin-3-*O*-rutinoside) was reported to have antioxidant capacity, and could upregulate the expression of antioxidant enzymes and enhance memory in transgenic mice as well as inhibit Aβ aggregation in vitro [111]. Several studies showed a neuroprotective effect of rutin in vitro and in vivo [111]. The administration of hesperidin improved learning and memory function by enhancing the recognition index in a transgenic mouse model [112]. It reduced the levels of Aβ_1–40_ and β- and γ-secretases in the hippocampus and the cortex of the brain of rats and modulated apoptosis via upregulating Bcl2 and downregulating Bax [112]. Chlorogenic acid can pass the blood-brain barrier and was reported to exhibit a protective effect on neuronal cells against oxidative damage and inhibit apoptotic regulators such as caspase-3 [113]. It inhibited AChE and BuChE in rat brain homogenates in vitro [113]. Chlorogenic acid was also found to prevent the overexpression of Aβ in human neuroblastoma cells and improve spatial learning and memory in an aging-mice model [113]. In addition, neoponcirin (isosakuranetin-7-*O*-rutinoside) was reported to possess a free radical scavenging activity and a neuroprotective effect against H_2_O_2_-induced toxicity through the activation of antioxidant defense enzymes and the inhibition of apoptosis [114]. Apigenin can cross the blood-brain barrier and was found to protect neurons against Aβ and inflammatory stimuli [115]. The treatment of a rat model of spinal cord injury with apigenin recovered neuronal function and showed anti-apoptotic effects, representing in a decrease in the expression levels of pro-apoptotic markers (Bax and caspase 3) and an elevation in the expression level of the anti-apoptotic protein Bcl-2 [116]. Luteolin was also reported to have a neuroprotective effect against apoptosis induced by 6-hydroxy-dopamine in rat PC12 cells [117].

Over the last 20 years, the search for pharmacologically relevant bioactivity in plants has often incorporated antioxidant testing as a way to preliminarily screen plant extracts and fractions. This approach has been based on the fact that oxidative stress is associated with many diseases. However, when dealing with a complex disease such as AD, which has multiple causes, relying on screening with antioxidant tests may lead to the rejection of extracts with other activities which may be relevant to AD. This idea was supported when a principal component analysis (PCA) was carried out to represent the broad spectrum of bioactivities exhibited by mint extracts [74]. Extracts of *M. australis* and *M. diemenica* showed a moderate antioxidant capacity [74] but a strong β-secretase inhibition activity [94]. The *M. requienii* extract had a high antioxidant capacity and Aβ-aggregation and caspase inhibition activities, but low AChE and moderate β-secretase inhibition activities, while *M.* × *piperita* var. *officinalis* had almost opposite responses [74,94]. The *M. spicata* var. *crispa* extract showed a high antioxidant capacity and histone deacetylase inhibition; however, it could not inhibit caspase to protect the cells from apoptotic death [74,94].

## 7. Variation in Phenolic Composition

When investigating the potential of plant extracts for treatment of diseases, it is important to also understand the factors that may affect the levels of bioactive compounds in the plant. The TPC of different extracts of several *Mentha* species and their hybrids showed a wide range of variability from 0.124 to 246.7 mg gallic acid equivalent (GAE)/g (Table S3), with an average of approximately 100 mg GAE/g [118,119]. For instance, the TPC of different extracts of *M. spicata* from India was 2.9–3.4 mg GAE/g [120] and 2.0–11.8 mg GAE/g [121]. Yi and Wetzstein [122] reported higher contents for *M.* × *piperita* and *M. spicata* cultivated in a greenhouse (76 and 63 mg GAE/g DW, respectively) than those grown in the field (39 and 55 mg GAE/g DW, respectively).

Several factors could influence the variation in TPC. While variation between species is expected, variation due to sample preparation was also observed. Different extraction techniques and conditions will yield different phenolic contents qualitatively and quantitatively for the same samples (Table 2) [123,124]. Bimakr et al. [125] found that different bioactive flavonoid compounds were obtained from *M. spicata* leaves by using conventional Soxhlet extraction (CSE) and supercritical carbon dioxide (SC-CO_2_) extraction at different extraction parameters such as temperature, pressure, and dynamic extraction time. CSE showed a higher crude extract yield than SC-CO_2_ extraction; however, the SC-CO_2_ extraction contained more main flavonoid compounds at a higher concentration than the CSE. Extraction using an accelerated solvent extractor, by hydrodistillation, or by heating at 60 °C gave a high TPC (115–247 mg GAE/g) [77,83,119]. Additionally, the polarity of extracting solvents can influence the solubility of chemical constituents and alter the analytical estimation of TPC from the same sample [126]. A 70% acetone extract of *M. arvensis* contained approximately 16 times the TPC of other solvents (70% ethanol, 70% methanol or water) [126]. Moreover, the drying technique may influence the estimation of TPC. Arslan et al. [127] found that drying *M.* × *piperita* in an oven at 50 °C or under direct sunlight at 20–30 °C or in a microwave oven resulted in approximately 53% lower, 16% higher, and 45% higher estimates, respectively, in the TPC as compared to a fresh sample. The increase in the estimated TPC could be due to the release of phenolic compounds from the matrix during the process, and drying might have facilitated the release of the bound compounds from the breakdown of cellular components, but the high temperature of oven drying might cause the thermal degradation of the phenolic compounds, which lowers the TPC of the oven-dried samples [127]. On the other hand, Riachi and De Maria [103] reported that drying peppermint at 60 °C in an oven led to a preserved total phenolic composition as compared to drying in shade, sunlight, or at 75 °C. The microwave oven drying at 900 W of *M. piperita* leaves yielded a higher TPC (406.7 mg GAE/100 g fresh weight (FW)) than drying using a hot-air oven at 80 °C (183.5 mg GAE/100 g FW), while the TPC of the fresh sample was 229 mg GAE/100 g FW [128]. The methanol extracts of *M. longifolia* produced higher TPCs by ultrasonic extraction than the 70% aqueous ethanol extracts, while the later gave better TPCs by Soxhlet [124]. Freeze-dried *M. aquatica* leaves contained a higher TPC (0.25 mg/g) than the microwave-dried samples (0.17 mg/g), while the latter contained a higher TPC than the oven-dried leaves (0.12 mg/g) [129].

Another factor that could explain the variations observed in the phytochemical content of plants is the different edaphoclimatic conditions to which they were subjected, which can influence the plant secondary metabolism [103]. Plants grown in temperate areas with extended hours of daylight contained a high content of phenolic acids [103]. As an example, *M. spicata* exposed to 16 h of daylight contained a higher content of rosmarinic acid than that exposed to 12 h [130]. Additionally, the main active constituent differed under different amounts of daylight in *M.* × *piperita* [103]. Peppermint grown under shorter photoperiods (8 h) had gardenin B as the main constituent, while that grown under longer photoperiods (16 h) had pebrellin [131].

Thus, the variables outlined above are important when considering the bioactivity of specific and batch to batch variations of plant-sourced compounds. The data reviewed here exemplifies one of the major challenges when considering multi-component plant extracts as therapeutics. Abundant evidence exists that mixed bioactives have synergistic and in some cases antagonistic effects, among the various components. Rigor in the characterization of plant extracts is essential, not only in the discovery phase, but importantly also (where the efficacy and absence of off-target effects are demonstrated) in the production of putative therapeutics.

## 8. Toxicity of *Mentha* Extracts

Despite the historical use of mints, toxicological studies on mint extracts are scarce. Treating neuroblastoma cells for 24 h with up to 1280 μg/mL 50% aqueous methanol extracts of *M. australis*, *M. diemenica*, *M. spicata*, *M.* × *piperita*, *M.* × *piperita* var. *officinalis*, or *M. requienii* had no toxic effect on the cell viability [94]. Exposure to 0.54, 5.4, and 53.8 mg/mL aqueous extract of *M.* × *piperita* for 24 h reduced the viability of *Tetrahymena pyriformi* cells in a dose-dependent manner [132]. The acute oral administration of 1000 mg/kg aqueous extract of *M. spicata* did not cause mortality, neurological alterations in rats, or changes in their normal behavior [133]. A histological analysis revealed no toxic effects at the hepatic or renal levels [133]. On the other hand, the chronic administration of 20 g/L *M. piperita* tea to rats (drinking at all times) during 30 days did not show signs of nephrotoxicity but induced hepatic damage and hormonal changes; however, 20 or 40 g/L *M. spicata* tea changed both the kidney and liver functions as well as the hormonal levels [134,135,136]. Considering the proportion of a human body weight to that of a rat, Akdogan, Kilinç, Oncu, Karaoz and Delibas [134] precluded a risk of nephrotoxicity of *M. spicata* tea. Neves [132] recommended a moderate human intake of *M.* × *piperita*, and Caro, Rivera, Ocampo, Franco and Salas [133] indicated the safety of *M. spicata*. Although the extensive use of *Mentha* extracts as herbal medicines implies their safety, the further evaluation of their potential adverse effects is essential.

## 9. Conclusions and Perspectives

The lack of a proper treatment or a prophylaxis for AD are drivers for further discovery and innovation, especially with the increasing number of patients suffering with AD. The exact etiology of AD is complicated and still poorly understood, with multiple factors contributing to neuronal cell death, making finding the way to a therapeutic approach challenging. With this in mind, it is worthwhile addressing various factors while searching for a potential treatment. The failure of recent clinical trials for synthetic compounds due to toxicity or worsening cognition suggests the need for an alternative approach. The ethnomedicinal value of *Mentha* points to its potential role in neurological conditions. The phenolic extracts and phenolic compounds present in *Mentha* species showed beneficial effects on the CNS. Many *Mentha* taxa had bioactivities linked to the different pathophysiological events of AD. They have a strong antioxidant capacity. They were able to inhibit the enzymes AChE, BuChE, HDAC, and β-secretase, meaning that they can modulate acetylcholine degradation, histone acetylation, and the formation of abnormal proteins. They also inhibited Aβ-accumulation and interfered with the pathological accumulation of tau through inhibiting GSK-3β and CK-1δ. Extracts from some taxa reduced ROS generation, exerted a neuroprotective effect against H_2_O_2_-induced oxidative stress, and enhanced the antioxidant enzymes in various cell lines. They could protect cells from apoptotic damage through modulating apoptotic regulators: suppressing caspases and pro-apoptotic proteins and enhancing anti-apoptotic proteins. Moreover, extracts from some taxa improved learning and cognition in mice and AD patients.

However, minimal research has been conducted that may indicate promising pathways towards developing either extracts from *Mentha* or in the discovery of specific compounds that may be refined and improved as bases for therapeutics for AD. The further explanation of the underlying molecular mechanism is still required. The determination of the pharmacokinetics, pharmacodynamics, bioavailability, and route of administration of mint extracts might provide further insight into the use of mint in food for brain health. The toxicity and ability of mint extracts to cross the blood-brain barrier to reach the neurons in the brain is an area that needs to be explored. The safety and efficacy still need to be assessed. Drinking mint tea or adding mint in food preparations is useful for the stomach but might be useful for brain health as well. How much is needed to exert its action is still to be discovered. *Mentha* can provide a promising source for AD medications as crude extracts or through the identification of novel bioactive compounds. The discovery and development of such components as a basis of pharmaceutical preparations is to be addressed. Instead of combining a number of agents to treat such a multifactorial disorder, which might not be practicable, *Mentha* extracts may have the potential to target multiple mechanisms of AD.

## Figures and Tables

**Table 1:** Chemical composition of some *Mentha* extracts that showed activities relevant to CNS, oxidative stress and AD.

Plant ^1^	Extraction Solvent	Chemical Composition	References
*M. aquatica*	50% aqueous methanol	danshensu, coumaroylquinic acid, caffeic acid, rosmarinic acid, salvianolic acid J, luteolin-7-*O*-diglucuronide, eriocitrin, apigenin-7-*O*-diglucuronide, luteolin-7-*O*-rutinoside, luteolin-7-*O*-glucuronide, apigenin-7-*O*-rutinoside, hesperidin, apigenin-7-*O*-glucuronide	[74]
	70% aqueous ethanol	naringenin	[70]
*M. arvensis*	50% aqueous methanol	chlorogenic acid, salvianolic acid I/J, rosmarinic acid, hesperidin	[74]
	80% aqueous methanol	linarin	[101]
*M. australis*	50% aqueous methanol	danshensu, caftaric acid, chlorogenic acid, lithospermic acid, rosmarinic acid, narirutin, isosakuranetin, linarin, neoponcirin	[74]
*M. diemenica*	50% aqueous methanol	danshensu, lithospermic acid, salvianolic acid A/H/I, rosmarinic acid, luteolin-7-*O*-diglucuronide, luteolin-7-*O*-glucuronide, apigenin-7-*O*-rutinoside, linarin	[74]
*M. gentilis*	50% aqueous methanol	rosmarinic acid, luteolin-7-*O*-rutinoside, apigenin-7-*O*-rutinoside, hesperidin, neoponcirin, acacetin-7-*O*-(6″-*O*-acetyl)-glucosylrhamnosylglucoside, linarin	[74]
*M. longifolia*	Ethanol	rosmarinic acid, chlorogenic acid, caffeic acid	[54]
*Mentha longifolia* var. *calliantha*	Ethanol or water	gallic acid, protocatechuic acid, *p*-hydroxybenzoic acid, chlorogenic acid, caffeic acid, syringic acid, vanillin, *p*-coumaric acid, sinapic acid, *o*-coumaric acid, rutin	[93]
*M. pulegium*	50% aqueous methanol	danshensu, caftaric acid, caffeic acid, chicoric acid, salvianolic acid B isomer, rosmarinic acid, salvianolic acid C, luteolin-7-*O*-diglucuronide, luteolin-7-*O*-rutinoside, luteolin-7-*O*-glucuronide, apigenin-7-*O*-glucuronide	[74]
	Ethanol	rosmarinic acid, chlorogenic acid, caffeic acid	[54]
*M. requienii*	50% aqueous methanol	danshensu, caftaric acid, chlorogenic acid, caffeic acid, chicoric acid, salvianolic acid B isomer, rosmarinic acid, salvianolic acid C, rutin	[74]
*M. spicata*	50% aqueous methanol	danshensu, chlorogenic acid, caffeic acid, rosmarinic acid, salvianolic acid B/C, luteolin-7-*O*-diglucuronide, eriocitrin, apigenin-7-*O*-diglucuronide, luteolin-7-*O*-rutinoside, luteolin-7-*O*-glucuronide, narirutin, apigenin-7-*O*-glucuronide, neoponcirin	[74]
*M. spicata* var. *crispa*	50% aqueous methanol	danshensu, caftaric acid, chlorogenic acid, caffeic acid, rosmarinic acid, salvianolic acid J, luteolin-7-*O*-rutinoside, luteolin-7-*O*-glucuronide, apigenin-7-*O*-rutinoside, hesperidin, neoponcirin	[74]
*M. suaveolens*	50% aqueous methanol	danshensu, coumaroylquinic acid, chlorogenic acid, caffeic acid, rosmarinic acid, salvianolic acid B/J, luteolin-7-*O*-rutinoside, luteolin-7-*O*-glucuronide, hesperidin	[74]
*M. suaveolens* var. *variegate*	50% aqueous methanol	danshensu, coumaroylquinic acid, chlorogenic acid, caffeic acid, rosmarinic acid, salvianolic acid A, luteolin-7-*O*-rutinoside, luteolin-7-*O*-glucuronide	[74]
*M. viridis*	50% aqueous methanol	danshensu, caffeic acid, salvianolic acid F/J, rosmarinic acid, eriocitrin, luteolin-7-*O*-rutinoside, luteolin-7-*O*-glucuronide, narirutin, apigenin-7-*O*-rutinoside, hesperidin, neoponcirin	[74]
*M.* × *niliaca*	50% aqueous methanol	danshensu, rosmarinic acid, salvianolic acid B/E, eriocitrin, luteolin-7-*O*-rutinoside, luteolin-7-*O*-glucuronide, narirutin, apigenin-7-*O*-rutinoside, hesperidin, neoponcirin	[74]
*M.* × *piperita*	50% aqueous methanol	danshensu, caftaric acid, chlorogenic acid, caffeic acid, salvianolic acid F/J, rosmarinic acid, luteolin-7-*O*-rutinoside, luteolin-7-*O*-glucuronide, narirutin, hesperidin, neoponcirin	[74]
	Ethanol	rosmarinic acid, chlorogenic acid, caffeic acid, ferulic acid	[54]
	70% aqueous methanol	luteolin diglucuronide, eriocitrin, luteolin rutinoside, salvianolic acid H/I/J, luteolin-7-*O*-glucuronide	[91]
*M.* × *piperita* var. *citrata*	50% aqueous methanol	rosmarinic acid, salvianolic acid J, apigenin-7-*O*-diglucuronide, eriocitrin, luteolin-7-*O*-diglucuronide, luteolin-7-*O*-rutinoside, luteolin-7-*O*-glucuronide, apigenin-7-*O*-rutinoside, narirutin, apigenin-7-*O*-glucuronide, hesperidin	[74]
*M.* × *piperita* var. *officinalis*	50% aqueous methanol	danshensu, coumaroylquinic acid, chlorogenic acid, salvianolic acid B/F, rosmarinic acid, luteolin-7-*O*-diglucuronide, luteolin-7-*O*-rutinoside, narirutin, linarin, hesperidin, apigenin-7-*O*-rutinoside, neoponcirin	[74]
*M.* × *piperita* f. *citrata* ʻChocolate’	50% aqueous methanol	chlorogenic acid, caffeic acid, rosmarinic acid, salvianolic acid B, luteolin-7-*O*-diglucuronide, eriocitrin, luteolin-7-*O*-rutinoside, apigenin-7-*O*-diglucuronide, apigenin-7-*O*-glucuronide, luteolin-7-*O*-glucuronide, apigenin-7-*O*-rutinoside, hesperidin	[74]
*M.* × *piperita* f. *citrata* ʻBasil’	50% aqueous methanol	danshensu, rosmarinic acid, salvianolic acid B, luteolin-7-*O*-diglucuronide, eriocitrin, apigenin-7-*O*-diglucuronide, luteolin-7-*O*-rutinoside, luteolin-7-*O*-glucuronide, apigenin-7-*O*-rutinoside, hesperidin, apigenin-7-*O*-glucuronide, neoponcirin	[74]

^1^ Taxonomy as per original published articles.

**Table 2:** Effect of extraction and/or drying methods on the phenolic content.

Extraction/Drying Method	Advantages	Disadvantages	References
conventional Soxhlet extraction	↑ crude extract yield Simple	↓ flavonoids at ↓ concentration time and solvent consuming not suitable for thermo-sensitive compounds	[125]
supercritical carbon dioxide extraction	↑ flavonoids at ↑ concentration extract selective soluble components	↓ crude extract yield CO_2_ is not suitable for extracting polar compounds	[125]
accelerated solvent extractor	↑ TPC ^3^		[77]
hydrodistillation extraction	↑ TPC		[83]
ultrasonic methanol extraction	↑ TPC	methanol is highly restricted in food or preservative industry	[124]
Soxhlet 70% ethanol extraction	↑ TPC		[124]
extraction at 60 °C	↑ TPC		[119]
70% acetone extraction	↑ TPC		[126]
ultrasonic 70% ethanol extraction	↓ TPC		[124]
70% ethanol extraction	↓ TPC		[126]
70% methanol extraction	↓ TPC		[126]
water extraction	↓ TPC		[126]
freeze-drying 5 min. at 60 °C	↑ TPC	↑ cost and time consuming	[129]
microwave oven drying	↑ TPC shortens drying time		[127,128]
direct sunlight drying at 20–30 °C	↑ TPC	samples get polluted, time consuming	[127]
oven drying at 50 °C	↓ TPC	not suitable for thermo-sensitive compounds	[127]
oven drying at 80 °C	↓ TPC	time consuming	[128]

↑ Content increased, ↓ Content decreased, ^3^ Total phenolic content.

## Supplementary Materials

The following are available online at https://www.mdpi.com/2076-3921/9/7/631/s1: Table S1: Ethnomedicinal uses of different *Mentha* taxa. Table S2: Chemical composition and bioactivities of different *Mentha* taxa. Table S3: Reported total phenolic content (TPC) for different *Mentha* taxa. Figure S1: Chemical structures of the main non-phenolic compounds reported in *Mentha* species. Figure S2: Chemical structures of the main phenolic compounds reported in *Mentha* species.

## References

[1] Korolev I.O. (2014). Alzheimer’s disease: A clinical and basic science review. Med. Stud. Res. J..

[2] Long J.M., Holtzman D.M. (2019). Alzheimer disease: An update on pathobiology and treatment strategies. Cell.

[3] Alzheimer’s Disease International (2019). World Alzheimer Report 2019: Attitudes to Dementia.

[4] (2019). 2019 Alzheimer’s disease facts and figures. Alzheimer’s Dement..

[5] Dementia Australia Home Page. https://www.dementia.org.au/statistics.

[6] Abyad A. (2015). Alzheimer’s in the Middle East. JSM Alzheimers Dis. Relat. Dement..

[7] Farrag A.-K., Farwiz H.M., Khedr E.H., Mahfouz R.M., Omran S.M. (1998). Prevalence of Alzheimer’s disease and other dementing disorders: Assiut-Upper Egypt study. Dement. Geriatr. Cogn. Disord..

[8] Zaitoun A.M., Sarhan A.-A.M.M.M., Selim A.M., Mousa G.R. (2008). Epidemiological study of dementia after retirement. Egypt. J. Neurol. Neurosurg..

[9] Jia L., Quan M., Fu Y., Zhao T., Li Y., Wei C., Tang Y., Qin Q., Wang F., Qiao Y. (2020). Dementia in China: Epidemiology, clinical management, and research advances. Lancet Neurol..

[10] Alzheimer Society Canada Home Page. https://alzheimer.ca/en/Home/About-dementia/What-is-dementia/Dementia-numbers.

[11] Rizzi L., Rosset I., Roriz-Cruz M. (2014). Global epidemiology of dementia: Alzheimer’s and vascular types. Biomed Res. Int..

[12] Obied H.K., Prenzler P.D., Omar S.H., Ismael R., Servili M., Esposto S., Taticchi A., Selvaggini R., Urbani S. (2012). Pharmacology of olive biophenols. Advances in Molecular Toxicology.

[13] Désiré L., Bourdin J., Loiseau N., Peillon H., Picard V., De Oliveira C., Bachelot F., Leblond B., Taverne T., Beausoleil E. (2005). RAC1 inhibition targets amyloid precursor protein processing by γ-secretase and decreases Aβ production in vitro and in vivo. J. Biol. Chem..

[14] Chen Y., Fu A.K.Y., Ip N.Y. (2019). Synaptic dysfunction in Alzheimer’s disease: Mechanisms and therapeutic strategies. Pharmacol. Ther..

[15] Panza F., Lozupone M., Logroscino G., Imbimbo B.P. (2019). A critical appraisal of amyloid-β-targeting therapies for Alzheimer disease. Nat. Rev. Neurol..

[16] Ullah R., Khan M., Shah S.A., Saeed K., Kim M.O. (2019). Natural antioxidant anthocyanins—A hidden therapeutic candidate in metabolic disorders with major focus in neurodegeneration. Nutrients.

[17] Moneim A.E.A. (2015). Oxidant/antioxidant imbalance and the risk of Alzheimer’s disease. Curr. Alzheimer Res..

[18] Gunn A.P., Wong B.X., Johanssen T., Griffith J.C., Masters C.L., Bush A.I., Barnham K.J., Duce J.A., Cherny R.A. (2016). Amyloid-β peptide Aβ3pE-42 induces lipid peroxidation, membrane permeabilization, and calcium influx in neurons. J. Biol. Chem..

[19] Gwon A.R., Park J.S., Arumugam T.V., Kwon Y.K., Chan S.L., Kim S.H., Baik S.H., Yang S., Yun Y.K., Choi Y. (2012). Oxidative lipid modification of nicastrin enhances amyloidogenic γ-secretase activity in Alzheimer’s disease. Aging Cell.

[20] Greenough M.A., Camakaris J., Bush A.I. (2013). Metal dyshomeostasis and oxidative stress in Alzheimer’s disease. Neurochem. Int..

[21] Wyss-Coray T., Rogers J. (2012). Inflammation in Alzheimer disease—A brief review of the basic science and clinical literature. Cold Spring Harb. Perspect. Med..

[22] Xu K., Dai X.-L., Huang H.-C., Jiang Z.-F. (2011). Targeting HDACs: A promising therapy for Alzheimer’s disease. Oxidative Med. Cell. Longev..

[23] De Ruijter A.J., Van Gennip A.H., Caron H.N., Kemp S., Van Kuilenburg A.B. (2003). Histone deacetylases (HDACs): Characterization of the classical HDAC family. Biochem. J..

[24] Wojsiat J., Zoltowska K.M., Laskowska-Kaszub K., Wojda U. (2018). Oxidant/antioxidant imbalance in Alzheimer’s disease: Therapeutic and diagnostic prospects. Oxidative Med. Cell. Longev..

[25] Obulesu M., Lakshmi M.J. (2014). Apoptosis in Alzheimer’s disease: An understanding of the physiology, pathology and therapeutic avenues. Neurochem. Res..

[26] Rang H.P., Ritter J.M., Flower R.J., Henderson G. (2015). Rang & Dale’s Pharmacology.

[27] Portt L., Norman G., Clapp C., Greenwood M., Greenwood M.T. (2011). Anti-apoptosis and cell survival: A review. Biochim. Biophys. Acta (BBA) Mol. Cell Res..

[28] Rohn T.T., Head E. (2008). Caspase activation in Alzheimer’s disease: Early to rise and late to bed. Rev. Neurosci..

[29] Shimohama S. (2000). Apoptosis in Alzheimer’s disease—An update. Apoptosis.

[30] Howells C.C., Baumann W.T., Samuels D.C., Finkielstein C.V. (2011). The Bcl-2-associated death promoter (BAD) lowers the threshold at which the Bcl-2-interacting domain death agonist (BID) triggers mitochondria disintegration. J. Theor. Biol..

[31] Li J., Li W., Jiang Z.-G., Ghanbari H.A. (2013). Oxidative stress and neurodegenerative disorders. Int. J. Mol. Sci..

[32] Nagai H., Noguchi T., Takeda K., Ichijo H. (2007). Pathophysiological roles of ASK1-MAP kinase signaling pathways. J. Biochem. Mol. Biol..

[33] Tan Y., Ruan H., Demeter M.R., Comb M.J. (1999). p90^RSK^ blocks Bad-mediated cell death via a protein kinase C-dependent pathway. J. Biol. Chem..

[34] Lu Z., Xu S. (2006). ERK1/2 MAP kinases in cell survival and apoptosis. IUBMB Life.

[35] Song J., Park K.A., Lee W.T., Lee J.E. (2014). Apoptosis signal regulating kinase 1 (ASK1): Potential as a therapeutic target for Alzheimer’s disease. Int. J. Mol. Sci..

[36] Latimer H.R., Veal E.A. (2016). Peroxiredoxins in regulation of MAPK signalling pathways; sensors and barriers to signal transduction. Mol. Cells.

[37] Hanschmann E.-M., Godoy J.R., Berndt C., Hudemann C., Lillig C.H. (2013). Thioredoxins, glutaredoxins, and peroxiredoxins—Molecular mechanisms and health significance: From cofactors to antioxidants to redox signaling. Antioxid. Redox Signal..

[38] Andoh T., Chock P.B., Chiueh C.C. (2002). The roles of thioredoxin in protection against oxidative stress-induced apoptosis in SH-SY5Y cells. J. Biol. Chem..

[39] Zhang R., Al-Lamki R., Bai L., Streb J.W., Miano J.M., Bradley J., Min W. (2004). Thioredoxin-2 inhibits mitochondria-located ASK1-mediated apoptosis in a JNK-independent manner. Circ. Res..

[40] Akterin S., Cowburn R.F., Miranda-Vizuete A., Jiménez A., Bogdanovic N., Winblad B., Cedazo-Minguez A. (2006). Involvement of glutaredoxin-1 and thioredoxin-1 in β-amyloid toxicity and Alzheimer’s disease. Cell Death Differ..

[41] Di Domenico F., Sultana R., Tiu G.F., Scheff N.N., Perluigi M., Cini C., Butterfield D.A. (2010). Protein levels of heat shock proteins 27, 32, 60, 70, 90 and thioredoxin-1 in amnestic mild cognitive impairment: An investigation on the role of cellular stress response in the progression of Alzheimer disease. Brain Res..

[42] Lovell M.A., Xie C., Gabbita S.P., Markesbery W.R. (2000). Decreased thioredoxin and increased thioredoxin reductase levels in Alzheimer’s disease brain. Free Radic. Biol. Med..

[43] Jarvis R.M., Hughes S.M., Ledgerwood E.C. (2012). Peroxiredoxin 1 functions as a signal peroxidase to receive, transduce, and transmit peroxide signals in mammalian cells. Free Radic. Biol. Med..

[44] Li B., Ishii T., Tan C.P., Soh J.-W., Goff S.P. (2002). Pathways of induction of peroxiredoxin I expression in osteoblasts: Roles of p38 mitogen-activated protein kinase and protein kinase C. J. Biol. Chem..

[45] Krapfenbauer K., Engidawork E., Cairns N., Fountoulakis M., Lubec G. (2003). Aberrant expression of peroxiredoxin subtypes in neurodegenerative disorders. Brain Res..

[46] Cumming R.C., Dargusch R., Fischer W.H., Schubert D. (2007). Increase in expression levels and resistance to sulfhydryl oxidation of peroxiredoxin isoforms in amyloid β-resistant nerve cells. J. Biol. Chem..

[47] Kim S., Fountoulakis M., Cairns N., Lubec G. (2001). Protein levels of human peroxiredoxin subtypes in brains of patients with Alzheimer’s disease and Down syndrome. Protein Expression in Down Syndrome Brain.

[48] Pae H.-O., Kim E.-C., Chung H.-T. (2008). Integrative survival response evoked by heme oxygenase-1 and heme metabolites. J. Clin. Biochem. Nutr..

[49] Mo L., Yang C., Gu M., Zheng D., Lin L., Wang X., Lan A., Hu F., Feng J. (2012). PI3K/Akt signaling pathway-induced heme oxygenase-1 upregulation mediates the adaptive cytoprotection of hydrogen peroxide preconditioning against oxidative injury in PC12 cells. Int. J. Mol. Med..

[50] Dennery P.A. (2014). Signaling function of heme oxygenase proteins. Antioxid. Redox Signal..

[51] Francis P.T., Palmer A.M., Snape M., Wilcock G.K. (1999). The cholinergic hypothesis of Alzheimer’s disease: A review of progress. J. Neurol. Neurosurg. Psychiatry.

[52] Adsersen A., Gauguin B., Gudiksen L., Jäger A.K. (2006). Screening of plants used in Danish folk medicine to treat memory dysfunction for acetylcholinesterase inhibitory activity. J. Ethnopharmacol..

[53] Imbimbo B.P., Watling M. (2019). Investigational BACE inhibitors for the treatment of Alzheimer’s disease. Expert Opin. Investig. Drugs.

[54] Vladimir-Knežević S., Blažeković B., Kindl M., Vladić J., Lower-Nedza A.D., Brantner A.H. (2014). Acetylcholinesterase inhibitory, antioxidant and phytochemical properties of selected medicinal plants of the Lamiaceae family. Molecules.

[55] Cicero A.F., Fogacci F., Banach M. (2018). Botanicals and phytochemicals active on cognitive decline: The clinical evidence. Pharmacol. Res..

[56] Shu Y.-Z. (1998). Recent natural products based drug development: A pharmaceutical industry perspective. J. Nat. Prod..

[57] Heinrich M., Lee Teoh H. (2004). Galanthamine from snowdrop—The development of a modern drug against Alzheimer’s disease from local Caucasian knowledge. J. Ethnopharmacol..

[58] Jones W.P., Chin Y.-W., Kinghorn A.D. (2006). The role of pharmacognosy in modern medicine and pharmacy. Curr. Drug Targets.

[59] Poison Control - National Capital Poison Center Home Page. https://www.poison.org/articles/tacrine-171.

[60] Declercq L.D., Vandenberghe R., Van Laere K., Verbruggen A., Bormans G. (2016). Drug development in Alzheimer’s disease: The contribution of PET and SPECT. Front. Pharmacol..

[61] Tucker A.O., Naczi R.F.C., Lawrence B.M. (2007). *Mentha*: An Overview of its Classification and Relationships. Mint: The Genus Mentha.

[62] Jabeen A., Guo B., Abbasi B.H., Shinwari Z.K., Mahmood T. (2012). Phylogenetics of selected *Mentha* species on the basis of rps8, rps11 and rps14 chloroplast genes. J. Med. Plants Res..

[63] Mahendran G., Rahman L.U. (2020). Ethnomedicinal, phytochemical and pharmacological updates on peppermint (*Mentha* × *piperita* L.)—A review. Phytother. Res..

[64] Peixoto I.T.A., Furletti V.F., Anibal P.C., Duarte M.C.T., Höfling J.F. (2010). Potential pharmacological and toxicological basis of the essential oil from *Mentha* spp.. Revista de Ciências Farmacêuticas Básica e Aplicada.

[65] Naghibi F., Mosaddegh M., Mohammadi Motamed M., Ghorbani A. (2010). Labiatae family in folk medicine in Iran: From ethnobotany to pharmacology. Iran. J. Pharm. Res..

[66] Karousou R., Balta M., Hanlidou E., Kokkini S. (2007). “Mints”, smells and traditional uses in Thessaloniki (Greece) and other Mediterranean countries. J. Ethnopharmacol..

[67] Mata A.T., Proença C., Ferreira A.R., Serralheiro M.L.M., Nogueira J.M.F., Araújo M.E.M. (2007). Antioxidant and antiacetylcholinesterase activities of five plants used as Portuguese food spices. Food Chem..

[68] Ayyobi H., Peyvast G.-A., Olfati J.-A. (2014). Effect of drying methods on essential oil yield, total phenol content and antioxidant capacity of peppermint and dill. Ratar. I Povrt..

[69] Fatiha B., Didier H., Naima G., Khodir M., Martin K., Léocadie K., Caroline S., Mohamed C., Pierre D. (2015). Phenolic composition, in vitro antioxidant effects and tyrosinase inhibitory activity of three Algerian *Mentha* species: *M. spicata* (L.), *M. pulegium* (L.) and *M. rotundifolia* (L.) Huds (Lamiaceae). Ind. Crop. Prod..

[70] Olsen H.T., Stafford G.I., van Staden J., Christensen S.B., Jäger A.K. (2008). Isolation of the MAO-inhibitor naringenin from *Mentha aquatica* L.. J. Ethnopharmacol..

[71] Biswas N.N., Saha S., Ali M. (2014). Antioxidant, antimicrobial, cytotoxic and analgesic activities of ethanolic extract of *Mentha arvensis* L.. Asian Pac. J. Trop. Biomed..

[72] Kapp K. (2015). Polyphenolic and Essential Oil Composition of Mentha and Their Antimicrobial Effect. Ph.D. Thesis.

[73] Brahmi F., Khodir M., Mohamed C., Pierre D., El-Shemy H. (2017). Chemical composition and biological activities of *Mentha* species. Aromatic and Medicinal Plants-Back to Nature.

[74] Hanafy D.M., Prenzler P.D., Burrows G.E., Ryan D., Nielsen S., El Sawi S.A., El Alfy T.S., Abdelrahman E.H., Obied H.K. (2017). Biophenols of mints: Antioxidant, acetylcholinesterase, butyrylcholinesterase and histone deacetylase inhibition activities targeting Alzheimer’s disease treatment. J. Funct. Foods.

[75] Salin O., Tormakangas L., Leinonen M., Saario E., Hagstrom M., Ketola R.A., Saikku P., Vuorela H., Vuorela P.M. (2011). Corn mint (*Mentha arvensis*) extract diminishes acute *Chlamydia pneumoniae* infection in vitro and in vivo. J. Agric. Food Chem..

[76] Kosar M., Dorman H.D., Can Baser K.H., Hiltunen R. (2004). Screening of free radical scavenging compounds in water extracts of *Mentha* samples using a postcolumn derivatization method. J. Agric. Food Chem..

[77] Stagos D., Portesis N., Spanou C., Mossialos D., Aligiannis N., Chaita E., Panagoulis C., Reri E., Skaltsounis L., Tsatsakis A.M. (2012). Correlation of total polyphenolic content with antioxidant and antibacterial activity of 24 extracts from Greek domestic Lamiaceae species. Food Chem. Toxicol..

[78] Conforti F., Ioele G., Statti G.A., Marrelli M., Ragno G., Menichini F. (2008). Antiproliferative activity against human tumor cell lines and toxicity test on Mediterranean dietary plants. Food Chem. Toxicol..

[79] Conforti F., Sosa S., Marrelli M., Menichini F., Statti G.A., Uzunov D., Tubaro A., Menichini F., Loggia R.D. (2008). In vivo anti-inflammatory and in vitro antioxidant activities of Mediterranean dietary plants. J. Ethnopharmacol..

[80] Baris O., Karadayi M., Yanmis D., Guvenalp Z., Bal T., Gulluce M. (2011). Isolation of 3 flavonoids from *Mentha longifolia* (L.) Hudson subsp. *longifolia* and determination of their genotoxic potentials by using the *E. coli* WP2 test system. J. Food Sci..

[81] Orhan F., Barış Ö., Yanmış D., Bal T., Güvenalp Z., Güllüce M. (2012). Isolation of some luteolin derivatives from *Mentha longifolia* (L.) Hudson subsp. *longifolia* and determination of their genotoxic potencies. Food Chem..

[82] Kapp K., Hakala E., Orav A., Pohjala L., Vuorela P., Püssa T., Vuorela H., Raal A. (2013). Commercial peppermint (*Mentha* × *piperita* L.) teas: Antichlamydial effect and polyphenolic composition. Food Res. Int..

[83] Dorman H.D., Kosar M., Kahlos K., Holm Y., Hiltunen R. (2003). Antioxidant properties and composition of aqueous extracts from *Mentha* species, hybrids, varieties, and cultivars. J. Agric. Food Chem..

[84] Ferreira A., Proenca C., Serralheiro M.L.M., Araujo M.E.M. (2006). The in vitro screening for acetylcholinesterase inhibition and antioxidant activity of medicinal plants from Portugal. J. Ethnopharmacol..

[85] Jäger A.K., Almqvist J.P., Vangsøe S.A.K., Stafford G.I., Adsersen A., Van Staden J. (2007). Compounds from *Mentha aquatica* with affinity to the GABA-benzodiazepine receptor. S. Afr. J. Bot..

[86] Moss M., Hewitt S., Moss L., Wesnes K. (2008). Modulation of cognitive performance and mood by aromas of peppermint and ylang-ylang. Int. J. Neurosci..

[87] Kim Y.C. (2010). Neuroprotective phenolics in medicinal plants. Arch. Pharmacal Res..

[88] Sabogal-Guáqueta A.M., Muñoz-Manco J.I., Ramírez-Pineda J.R., Lamprea-Rodriguez M., Osorio E., Cardona-Gómez G.P. (2015). The flavonoid quercetin ameliorates Alzheimer’s disease pathology and protects cognitive and emotional function in aged triple transgenic Alzheimer’s disease model mice. Neuropharmacology.

[89] Phan H.T., Samarat K., Takamura Y., Azo-Oussou A.F., Nakazono Y., Vestergaard M.d.C. (2019). Polyphenols modulate Alzheimer’s amyloid beta aggregation in a structure-dependent manner. Nutrients.

[90] López V., Martín S., Gómez--Serranillos M.P., Carretero M.E., Jäger A.K., Calvo M.I. (2010). Neuroprotective and neurochemical properties of mint extracts. Phytother. Res..

[91] Gürbüz P., Martinez A., Pérez C., Martínez-González L., Göger F., Ayran İ. (2019). Potential anti-Alzheimer effects of selected Lamiaceae plants through polypharmacology on glycogen synthase kinase-3β, β-secretase, and casein kinase 1δ. Ind. Crop. Prod..

[92] Benabdallah A., Rahmoune C., Boumendjel M., Aissi O., Messaoud C. (2016). Total phenolic content and antioxidant activity of six wild *Mentha* species (Lamiaceae) from northeast of Algeria. Asian Pac. J. Trop. Biomed..

[93] Bahadori M.B., Zengin G., Bahadori S., Dinparast L., Movahhedin N. (2018). Phenolic composition and functional properties of wild mint (*Mentha longifolia* var. *calliantha* (Stapf) Briq.). Int. J. Food Prop..

[94] Hanafy D.M., Prenzler P.D., Burrows G.E., Gurusinghe S., Thejer B.M., Obied H.K., Hill R.A. (2020). Neuroprotective activity of *Mentha* species on hydrogen peroxide-induced apoptosis in SH-SY5Y cells. Nutrients.

[95] Lee H., Yeom M., Shin S., Jeon K., Park D., Jung E. (2019). Protective effects of aqueous extract of *Mentha suaveolens* against oxidative stress-induced damages in human keratinocyte HaCaT cells. Evid. Based Complementary Altern. Med..

[96] Joshi H., Bhadania M. (2014). Evaluation of freeze dried extract of *Mentha piperita* in management of cognitive dysfunctions in mice. Alzheimers Dement..

[97] Hassan H.A., Hafez H.S., Goda M.S. (2013). *Mentha piperita* as a pivotal neuro-protective agent against gamma irradiation induced DNA fragmentation and apoptosis: *Mentha* extract as a neuroprotective against gamma irradiation. Cytotechnology.

[98] Ahmad M., Arshad H., Kalam N.A., Anshu M., Hasin A.M., Shadma W. (2012). Effect of the aqueous extract of *Mentha arvensis* on haloperidol induced catalepsy in albino mice. J. Clin. Diagn. Res.

[99] Farr S.A., Niehoff M.L., Ceddia M.A., Herrlinger K.A., Lewis B.J., Feng S., Welleford A., Butterfield D.A., Morley J.E. (2016). Effect of botanical extracts containing carnosic acid or rosmarinic acid on learning and memory in SAMP8 mice. Physiol. Behav..

[100] Herrlinger K.A., Nieman K.M., Sanoshy K.D., Fonseca B.A., Lasrado J.A., Schild A.L., Maki K.C., Wesnes K.A., Ceddia M.A. (2018). Spearmint extract improves working memory in men and women with age-associated memory impairment. J. Altern. Complementary Med..

[101] Oinonen P.P., Jokela J.K., Hatakka A.I., Vuorela P.M. (2006). Linarin, a selective acetylcholinesterase inhibitor from *Mentha arvensis*. Fitoterapia.

[102] Lou H., Fan P., Perez R.G., Lou H. (2011). Neuroprotective effects of linarin through activation of the PI3K/Akt pathway in amyloid-β-induced neuronal cell death. Bioorganic Med. Chem..

[103] Riachi L.G., De Maria C.A.B. (2015). Peppermint antioxidants revisited. Food Chem..

[104] Chien M.-Y., Chuang C.-H., Chern C.-M., Liou K.-T., Liu D.-Z., Hou Y.-C., Shen Y.-C. (2016). Salvianolic acid A alleviates ischemic brain injury through the inhibition of inflammation and apoptosis and the promotion of neurogenesis in mice. Free Radic. Biol. Med..

[105] Lee H.J., Cho H.-S., Park E., Kim S., Lee S.-Y., Kim C.-S., Kim D.K., Kim S.-J., Chun H.S. (2008). Rosmarinic acid protects human dopaminergic neuronal cells against hydrogen peroxide-induced apoptosis. Toxicology.

[106] Rong H., Liang Y., Niu Y. (2018). Rosmarinic acid attenuates β-amyloid-induced oxidative stress via Akt/GSK-3β/Fyn-mediated Nrf2 activation in PC12 cells. Free Radic. Biol. Med..

[107] Hamaguchi T., Ono K., Murase A., Yamada M. (2009). Phenolic compounds prevent Alzheimer’s pathology through different effects on the amyloid-β aggregation pathway. Am. J. Pathol..

[108] Bae H.J., Sowndhararajan K., Park H.-B., Kim S.-Y., Kim S., Kim D.H., Choi J.W., Jang D.S., Ryu J.H., Park S.J. (2019). Danshensu attenuates scopolamine and amyloid-β-induced cognitive impairments through the activation of PKA-CREB signaling in mice. Neurochem. Int..

[109] Zhou Y., Li W., Xu L., Chen L. (2011). In *Salvia miltiorrhiza*, phenolic acids possess protective properties against amyloid β-induced cytotoxicity, and tanshinones act as acetylcholinesterase inhibitors. Environ. Toxicol. Pharmacol..

[110] Chong C.-M., Zhou Z.-Y., Razmovski-Naumovski V., Cui G.-Z., Zhang L.-Q., Sa F., Hoi P.-M., Chan K., Lee S.M.-Y. (2013). Danshensu protects against 6-hydroxydopamine-induced damage of PC12 cells in vitro and dopaminergic neurons in zebrafish. Neurosci. Lett..

[111] Enogieru A.B., Haylett W., Hiss D.C., Bardien S., Ekpo O.E. (2018). Rutin as a potent antioxidant: Implications for neurodegenerative disorders. Oxidative Med. Cell. Longev..

[112] Hajialyani M., Hosein Farzaei M., Echeverría J., Nabavi S.M., Uriarte E., Sobarzo-Sánchez E. (2019). Hesperidin as a neuroprotective agent: A review of animal and clinical evidence. Molecules.

[113] Heitman E., Ingram D.K. (2017). Cognitive and neuroprotective effects of chlorogenic acid. Nutr. Neurosci..

[114] Yao Q., Lin M.-T., Zhu Y.-D., Xu H.-L., Zhao Y.-Z. (2018). Recent Trends in potential therapeutic applications of the dietary flavonoid didymin. Molecules.

[115] Dourado N.S., Souza C.d.S., de Almeida M.M.A., Bispo da Silva A., dos Santos B.L., Silva V.D.A., De Assis A.M., da Silva J.S., Souza D.O., Costa M.d.F.D. (2020). Neuroimmunomodulatory and neuroprotective effects of the flavonoid apigenin in in vitro models of neuroinflammation associated with Alzheimer’s disease. Front. Aging Neurosci..

[116] Zhang F., Li F., Chen G. (2014). Neuroprotective effect of apigenin in rats after contusive spinal cord injury. Neurol. Sci..

[117] Guo D.J., Li F., Yu P.H., Chan S.W. (2013). Neuroprotective effects of luteolin against apoptosis induced by 6-hydroxydopamine on rat pheochromocytoma PC12 cells. Pharm. Biol..

[118] Al-Juhaimi F., Ghafoor K. (2011). Total phenols and antioxidant activities of leaf and stem extracts from coriander, mint and parsley grown in Saudi Arabia. Pak. J. Bot..

[119] Benedec D., Vlase L., Oniga I., Mot A.C., Silaghi-Dumitrescu R., Hanganu D., Tiperciuc B., Crişan G. (2013). LC-MS analysis and antioxidant activity of phenolic compounds from two indigenous species of *Mentha*. Note I. Farmacia.

[120] Biswas A.K., Chatli M.K., Sahoo J. (2012). Antioxidant potential of curry (*Murraya koenigii* L.) and mint (*Mentha spicata*) leaf extracts and their effect on colour and oxidative stability of raw ground pork meat during refrigeration storage. Food Chem..

[121] Malik B., Sharma N.R., Soni G. (2013). Influence of agro-climatic conditions on antioxidant potential of *Mentha* species. J. Pharm. Res..

[122] Yi W., Wetzstein H.Y. (2010). Biochemical, biological and histological evaluation of some culinary and medicinal herbs grown under greenhouse and field conditions. J. Sci. Food Agric..

[123] Antolovich M., Prenzler P., Robards K., Ryan D. (2000). Sample preparation in the determination of phenolic compounds in fruits. Analyst.

[124] Patonay K., Szalontai H., Csugány J., Szabó-Hudák O., Kónya E.P., Németh É.Z. (2019). Comparison of extraction methods for the assessment of total polyphenol content and in vitro antioxidant capacity of horsemint (*Mentha longifolia* (L.) L.). J. Appl. Res. Med. Aromat. Plants.

[125] Bimakr M., Rahman R.A., Taip F.S., Ganjloo A., Salleh L.M., Selamat J., Hamid A., Zaidul I.S.M. (2011). Comparison of different extraction methods for the extraction of major bioactive flavonoid compounds from spearmint (*Mentha spicata* L.) leaves. Food Bioprod. Process..

[126] Sulaiman S.F., Sajak A.A.B., Ooi K.L., Seow E.W. (2011). Effect of solvents in extracting polyphenols and antioxidants of selected raw vegetables. J. Food Compos. Anal..

[127] Arslan D., Özcan M.M., Mengeş H.O. (2010). Evaluation of drying methods with respect to drying parameters, some nutritional and colour characteristics of peppermint (*Mentha* x *piperita* L.). Energy Convers. Manag..

[128] Hayat K. (2020). Impact of drying methods on the functional properties of peppermint (*Mentha piperita* L.) leaves. Sci. Lett..

[129] Safaiee P., Taghipour A., Vahdatkhoram F., Movagharnejad K. (2019). Extraction of phenolic compounds from *Mentha aquatica*: The effects of sonication time, temperature and drying method. Chem. Pap..

[130] Fletcher R.S., Slimmon T., Kott L.S. (2010). Environmental factors affecting the accumulation of rosmarinic acid in spearmint (*Mentha spicata* L.) and peppermint (*Mentha piperita* L.). Open Agric. J..

[131] Voirin B., Saunois A., Bayet C. (1994). Free flavonoid aglycones from *Mentha* × *piperita*: Developmental, chemotaxonomical and physiological aspects. Biochem. Syst. Ecol..

[132] Neves J.M. (2005). Concerns regarding the toxicity of *Mentha* x *piperita*. Ann. Med. Chem. Res..

[133] Caro D.C., Rivera D.E., Ocampo Y., Franco L.A., Salas R.D. (2018). Pharmacological evaluation of *Mentha spicata* L. and *Plantago major* L., medicinal plants used to treat anxiety and insomnia in Colombian Caribbean Coast. Evid Based Complement Altern. Med.

[134] Akdogan M., Kilinç I., Oncu M., Karaoz E., Delibas N. (2003). Investigation of biochemical and histopathological effects of *Mentha piperita* L. and *Mentha spicata* L. on kidney tissue in rats. Hum. Exp. Toxicol..

[135] Akdogan M., Ozguner M., Aydin G., Gokalp O. (2004). Investigation of biochemical and histopathological effects of *Mentha piperita* Labiatae and *Mentha spicata* Labiatae on liver tissue in rats. Hum. Exp. Toxicol..

[136] Akdogan M., Ozguner M., Kocak A., Oncu M., Cicek E. (2004). Effects of peppermint teas on plasma testosterone, follicle-stimulating hormone, and luteinizing hormone levels and testicular tissue in rats. Urology.

